# A sound mind in a sound body? The role of cooperation between medical specialists and patients with comorbid mental and somatic disorders

**DOI:** 10.1192/j.eurpsy.2021.2113

**Published:** 2021-08-13

**Authors:** K. Adamczewska, M. Bień, K. Dudzic, K. Krysta, M. Krzystanek

**Affiliations:** 1 Students` Scientific Association, Department Of Rehabilitation Psychiatry, Medical University of Silesia, Katowice, Poland; 2 Department Of Rehabilitation Psychiatry, Medical University of Silesia, Katowice, Poland

**Keywords:** schizophrénia, somatic disorders, comorbidity

## Abstract

**Introduction:**

Treatment of accompanying somatic disorders in patients with schizophrenia is a crucial issue, as those people die about 25 years earlier, compared with the general population. Moreover, premature death in this group of patients is more often caused by comorbidities than by suicide. It is worth emphasizing that cardiovascular disease itself in people with schizophrenia accounts for as much as 23% of causes of death, followed by suicides and drug toxicity. The paper presents a description of a 65-year-old patient diagnosed with schizophrenia, alcohol addiction, metabolic syndrome, and cardiac arrhythmia.

**Objectives:**

To determine the impact of cooperation between medical specialists and a psychiatric patient on the treatment effect.

**Methods:**

A case of a patient treated in a day ward is described. A literature search was made in the PubMed database.

**Results:**

A patient after exacerbations of mental illness, often preceded by a return to alcohol use, tends to discontinue both psychiatric drugs and those prescribed for somatic diseases. Due to the diagnosed atrial fibrillation, sudden discontinuation of cardiac medications significantly increases the risk of life-threatening somatic complications, including stroke.

**Conclusions:**

Diagnostic and therapeutic management in the treatment of psychiatric and somatic diseases with concurrent addiction to psychoactive substances requires interdisciplinary cooperation of medical specialists with the patient to achieve a successful outcome. Summarizing, in treatment, we must always look at the patient as a whole. Aside from caring for the mental state, the physical condition along with the possibility of cooperation on the part of the patient remains essential.

**Disclosure:**

No significant relationships.

